# Accelerometers can correctly count orthopaedic patients' early post‐operative steps while using walking aids

**DOI:** 10.1002/jeo2.70134

**Published:** 2025-01-03

**Authors:** Spiros Tsamassiotis, Michael Schwarze, Philipp Gehring, Roman F. Karkosch, Lars‐René Tücking, Ann‐Kathrin Einfeldt, Eike Jakubowitz

**Affiliations:** ^1^ Department of Orthopaedic Surgery Hannover Medical School, Diakovere Annastift Hannover Germany; ^2^ Department of Orthopaedic Surgery Hannover Medical School, Laboratory for Biomechanics and Biomaterials Hannover Germany

**Keywords:** accelerometer, accuracy, assistive device, early post‐operative period, orthopaedic rehabilitation, speed, step count, walking aid, wearable, weight‐bearing

## Abstract

**Purpose:**

Effective rehabilitation after orthopaedic surgery is critical. The early post‐operative phase is increasingly managed in outpatient settings, necessitating objective measures such as step counts to monitor rehabilitation progress. However, it remains unclear if commercially available wearables or accelerometers using simple algorithms can accurately count steps in early post‐operative conditions. We hypothesised that only accelerometers could accurately determine the number of steps under these conditions.

**Methods:**

This case series involved 20 healthy subjects, 7 female and 13 males, walking in a circle at varying speeds under partial loading with three different walking aids (forearm crutches, walking frame and rolling walker) and four wearables (Vivofit 4, Fenix 3HR, Fitbit Charge 3 and Omron HJ‐325) and one accelerometer (AX6) worn on the wrist, hip and ankle. The two‐point and modified three‐point gait patterns commonly used post‐operatively were simulated. The primary end point was the relative error (RE), defined as RE = (manual count − automated count)/manual count, of each wearable measurement compared to visual and video step counting, the gold standard.

**Results:**

The RE of AX6 and Fitbit was less than 0.1 for all walking aids except the rolling walker, with AX6 showing the lowest standard deviation (SD) compared to other wearables. Other wearables had significantly higher RE. Increased gait speed generally improved accuracy, reducing RE in most devices, except for the AX6, which showed the opposite trend. At 0.6 m/s, only AX6 achieved an RE below 0.1. The ankle was identified as the best measuring location.

**Conclusion:**

During the early post‐operative period, commercial wearables can only accurately count steps under specific conditions and should be used cautiously for monitoring steps in the early post‐operative phase. However, accelerometers with appropriate coding appear suitable for this purpose.

**Level of Evidence:**

Level III diagnostic study.

**Abbreviations**
IC: initial contact
MAPE: mean absolute percentage error
PROM: patient‐reported outcome measurement
RE: relative error
SD: standard deviation

## INTRODUCTION

Both the operation and subsequent rehabilitation phase are crucial for achieving good results after orthopaedic surgery [22, 64]. Rapid and sufficient post‐operative mobilisation is very important in this context. Early mobilisation of the patient plays a crucial role, particularly in fast‐track programmes that are used more frequently nowadays, partly because early mobilisation significantly reduces the risk of complications [24, 31]. Early mobilisation is also crucial in the management of fractures. For example, early mobilisation can significantly reduce mortality in geriatric patients [3]. However, the worldwide use of fast‐track programmes also means that patients are discharged sooner, so clinicians lose their influence on early mobilisation [29]. Therefore, simple, reliable and objectifiable monitoring parameters are needed to record the outpatient rehabilitation process correctly. A good and simple way to quantify the patient's early mobilisation level is step counting [23, 40]. Early post‐operative step count also has a strong influence on 1‐year outcome after lower extremity fractures [44]. However, medical pedometers and gait monitoring insoles are quite complicated to use, require their own separate software, and are very expensive. They are therefore not suitable for everyday clinical use or in an outpatient setting. Commercially available activity trackers, such as smart watches, are enjoying great popularity and are increasingly finding their way into everyday life [58]. They offer the user a variety of functions. One of the most frequently used features is fitness monitoring. The ease of use and the direct feedback on progress also make their use attractive for older patients [9, 50]. Therefore, commercial wearables are increasingly finding their way into clinical research with the prospect of being used in everyday clinical practice, not least because the early post‐operative phase of patients is increasingly being outsourced to the outpatient sector [1, 2, 25, 29]. However, commercial activity trackers and pedometers interpret the acceleration data using manufacturer‐specific detection algorithms, which are generally unknown to the user and are adapted to a healthy collective with a normal gait pattern. The step counts are often based on an acceleration threshold algorithm [12]. However, the gait pattern of patients in the early post‐operative phase is highly altered, particularly regarding gait speed, loading of the operated limb and performing specific gait patterns like the 2‐ or 3‐point gait [4, 30]. In addition, patients are usually dependent on various walking aids, which also alter gait [15, 30]. Yet this is not considered in the aforementioned detection algorithms, meaning that the number of steps displayed may differ significantly from the actual number of steps [30]. Accelerometers have the advantage over wearables in generating raw data. This raw data can then be analysed using an algorithm tailored to the conditions being assessed. From this point of view, the usability of wearables as opposed to accelerometers in this specific clinical context needs to be further investigated [16, 34, 41], as there is a paucity of data in the current literature regarding adequate step counting when walking aids are used in combination with reduced walking speed, partial weight‐bearing and performance of the two‐point and modified three‐point gait patterns. Consequently, the aim of this study was to investigate how precisely commercially available activity trackers and a low‐cost, small‐sized accelerometer can reflect the number of steps taken under the above‐mentioned simulated post‐operative conditions.

Therefore, we hypothesise that accelerometers, in contrast to commercial wearables, can accurately reflect the number of steps taken by orthopaedic patients in the early post‐operative phase and that commercial wearables should be used with caution for step counting in the early post‐operative phase of orthopaedic patients.

## MATERIALS AND METHODS

All procedures described in this study were approved by the Ethics Committee of Hannover Medical School (No. 9516).

### Subjects

Twenty medical students, 7 females and 13 males, from Hannover Medical School participated in the study. Participants were recruited from the Hannover Medical School community and surrounding areas through word‐of‐mouth. Inclusion criteria were subjects over 20 years of age with no known orthopaedic or neurological disease. Exclusion criteria were subjects over 35 years of age and underlying neurological or orthopaedic diseases. The average age was 25.1 years (standard deviation [SD] 2.2), and the average body mass index was 22.6 (SD 2.1). All participants signed a written informed consent.

### Instruments

**Figure 1:**
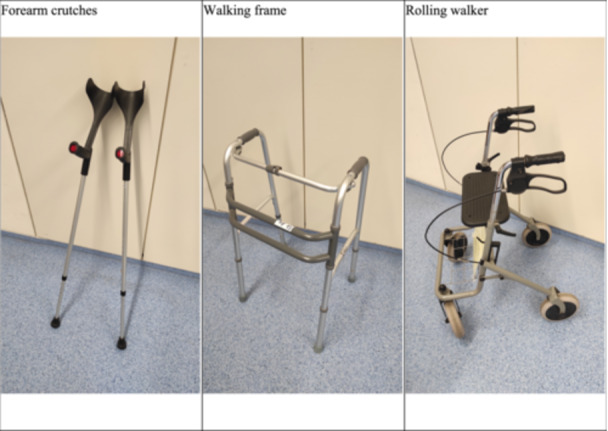
Included walking aids.

The activity trackers were selected based on their level of popularity in the population, their frequency of use in other studies, and their market price, as the financial aspect in terms of acquisition costs is often decisive for clinical use. The Vivofit 4 (Garmin), the Fenix 3HR (also Garmin), the Fitbit Charge 3 (Fitbit Inc.), the Omron Walking Style IV (Type HJ‐325, Omron Corporation), and the six‐axis accelerometer AX6 (Axivity Ltd.) were therefore used. The price ranged from approximately €35 (Omron) to €600 (Fenix). The walking aids used were height‐adjustable forearm crutches, a reciprocal walking frame and a rolling walker, which are all routinely used in the orthopaedic post‐operative setting (Figure 1). Furthermore, a rotating laser was used to project a line onto the floor, rotating in a circular path. The speed of the rotating laser line could be varied and was used to control the participants' walking speed.

### Procedure

A circle with a radius of 3 m was marked on the floor of the gait lab. The participants walked around the circle with forearm crutches, once in a modified three‐point and once in a two‐point gait, with the walking frame as well as with the rolling walker. All trackers and the accelerometer were attached one after the other to the wrist, hip and ankle. Each type of gait and each tracker position were tested at 0.6, 0.8 and 1 m/s gait speed. The duration of each speed trial was 2 min. The participants simulated an impaired leg after surgery during the trials. The subjects were free to choose which leg side served for this simulation and they were asked to load it with half their body weight. The partial load was introduced to them before trials using a scale (Type 877, Seca GmbH & Co. KG). All trackers and the accelerometer were always attached to the contralateral side, since reduced acceleration due to partial loading may produce false count values [13]. The participants wore their own sportswear and sports shoes. The steps measured by the activity trackers and the accelerometer were contrasted with visual step counting. The visual step count was cross‐checked with video recordings of the gait cycles to identify any errors that may have occurred during visual counting. A preliminary test found that almost no steps could be detected when using the rolling walker with the trackers positioned at the wrist. Besides, gait cycles with the reciprocal walking frame at 1 m/s were not measured, as this speed was almost impossible to realise with the walking frame, even for healthy subjects. The number of gait cycles was reduced by making twice the number of trackers available. Eight trackers were attached to the participants at the same time. This resulted in a total of 858 cycles and thus 1716 min of examination time.

### Accelerometer analysis

**Figure 2:**
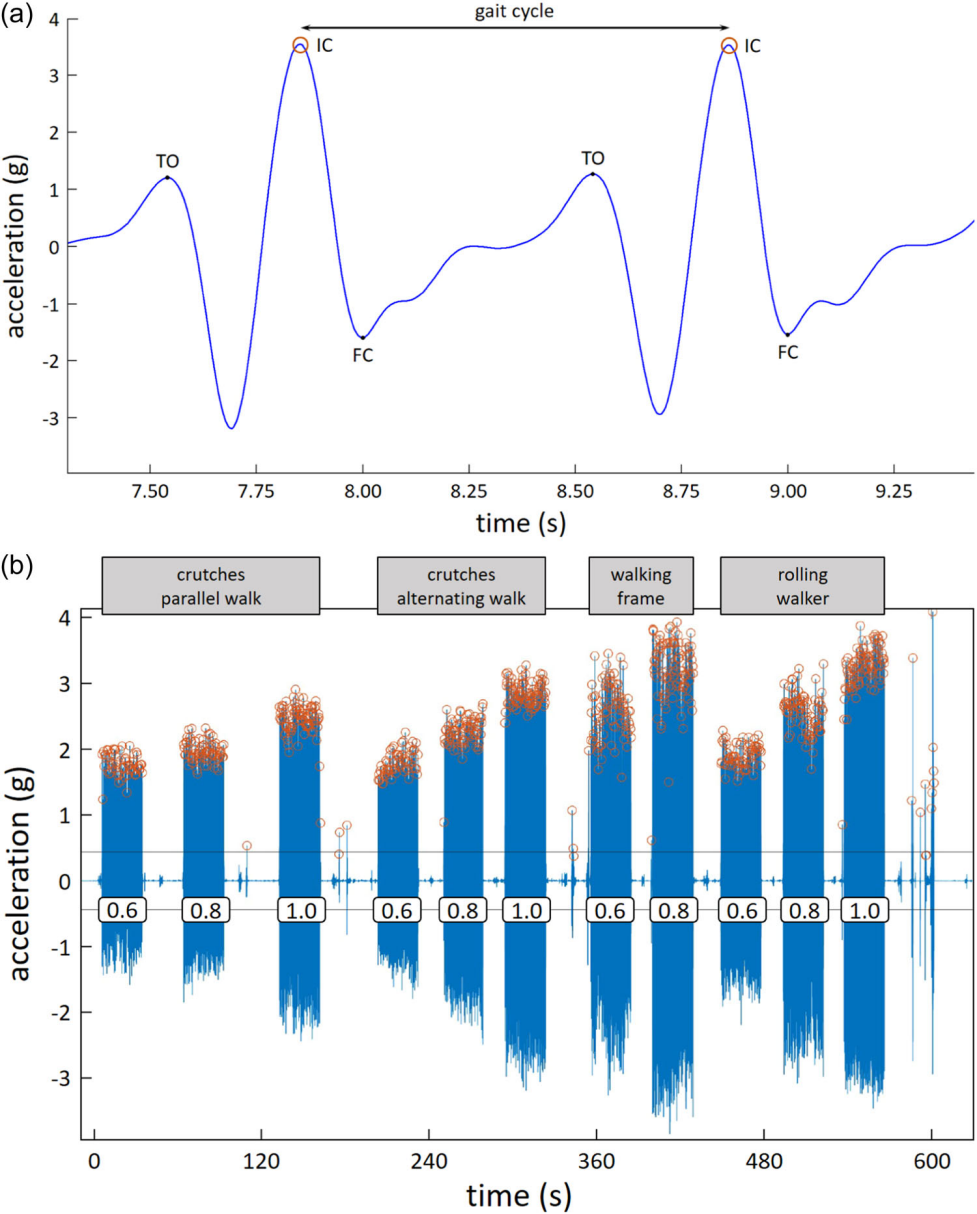
(a) Enlarged section of the signal from (b) of the step count measurement with the AX6 positioned at the ankle of subject no. 12 with the rolling walker and gait speed of 1 m/s. A gait cycle was delimited by two initial contacts (ICs). Other prominent step cycle points that would have been drawn were the toe‐off (TO) and the full contact (FC). (b) Processed raw signal from AX6 positioned at the ankle of participant no. 12 as a function of the respective walking aid (grey boxes at the top) and the respective walking speed (white boxes in the minus range in m/s). The brown circles indicate the steps identified by the algorithm (ICs from (a)).

Matlab software (R2021a, MathWorks Inc.) was used to analyse the post‐processed data from the AX6. Its sensor captures the acceleration within space with a 10 Hz sampling rate. As the steps in this direction are clearly distinguishable, only the acceleration data in the vertical direction of the accelerometer was analysed (Figure 2a). Data representing the initial contact (IC) of the foot during walking were then identified from the acceleration data as maximum values. Only signals of the extremity on which the sensor was attached could be discriminated. Therefore, gait cycles were counted, which were then doubled to the number of steps. The local maxima of the IC phase, which was above 70% of the standard deviation for the sensor position at the hip, and 80% of the standard deviation for the sensor position at the wrist and at the ankle, and had a minimum distance of 1 s, were determined. Single measurements were obtained by searching for areas where 30 of the marked maxima were located at a distance less than 3–3.5 sfrom each other. After determining such an area, the next 125 s were analysed separately in a new data set. Maxima with a minimum distance of 0.9 s and whose values were above 70% (hip) or 80% (wrist, ankle) of the SD of the single measurements were registered as ICs. Depending on the walking aid, a different sensor excursion resulted (Figure 2b). This was considered by determining the SD of the individual measurements. The selected limits (0.9 s, 70% and 80% of the SD) were determined iteratively; the smallest deviation from the visually counted number of steps was found in this threshold range.

### Statistical analysis

With a power analysis for a one‐sided *t* test for connected samples, a significance level of 0.05 and a power of 0.8 resulted in a minimum sample size of 19 subjects, which was rounded up to 20.

Data were analysed using R (version 4.3.0) [46]. A measurement's relative error (RE), defined as relerror = (manual_count‐automated_count)/manual_count of the individual measurement runs, was used for the primary evaluation. An RE of 0 is the result of an ideal measurement, while positive values indicate that the number of automatic steps counted is too low and vice versa. Mean and standard deviation were then calculated.

## RESULTS

The AX6 data of three participants could not be analysed due to a transmission error, meaning that only 17 test participants were analyzed for the AX6. One subject suffered a proximal humerus fracture in a private context and was not able to use walking aids during the study period so that only 19 subjects were analyzed for the HJ‐325.

**Figure 3:**
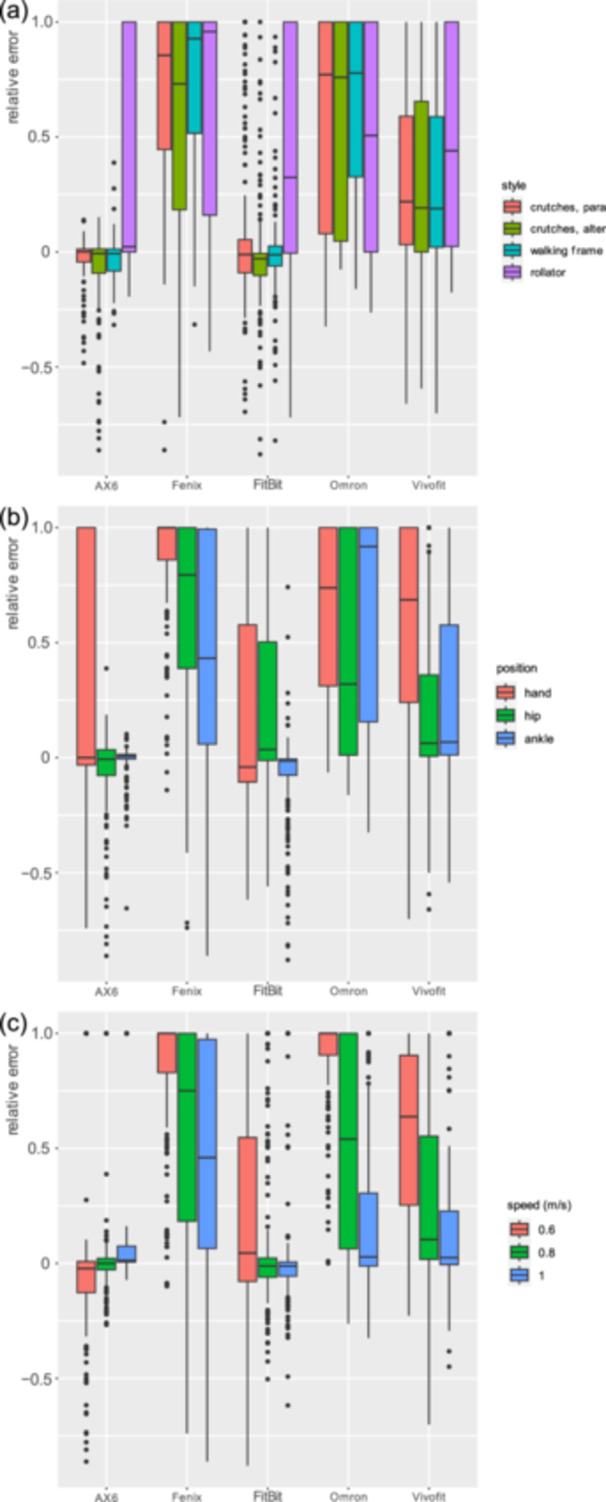
REs of each measurement unit in relation to (a) used walking aids, (b) the location of the measuring unit and (c) the walking speed. RE, relative error.

AX6 and Fitbit had the lowest RE regarding the walking aid or gait type with the crutches (Table 1, Figure 3). In modified three‐point gait with crutches (−0.03) and using the rolling walker (0.33), the AX6 generated a smaller RE than the Fitbit (0.05/0.42); in two‐point gait with crutches and with the reciprocal walking frame, the opposite was the case. The AX6 showed an RE of −0.08 and −0.03, and the Fitbit of −0.02 and 0.01. The AX6 always produced a smaller SD than the Fitbit. The Fenix watch had the largest RE, followed by the Omron, and Vivofit sensors.

**Table 1:** RE as mean and SD values for each sensor type and activity with gait aids.

	Activity							
	Crutches, mod. 3‐point gait		Crutches, 2‐point gait		Walking frame		Rolling walker	
Sensor	Mean	SD	Mean	SD	Mean	SD	Mean	SD
AX6	−0.03	0.10	−0.08	0.20	‐0.03	0.10	0.33	0.47
Fitbit	0.05	0.32	−0.02	0.24	0.01	0.25	0.42	0.48
Fenix	0.70	0.38	0.61	0.40	0.72	0.38	0.67	0.42
Omron	0.58	0.44	0.57	0.43	0.64	0.37	0.49	0.46
Vivofit	0.33	0.36	0.30	0.39	0.28	0.34	0.48	0.45

Abbreviations: RE, relative error; SD, standard deviation.

In terms of gait speed, at 0.6 m/s the AX6 delivered the smallest RE (−0.02), followed by Fitbit (0.21), Vivofit (0.57), Fenix (0.87) and Omron (0.92) (Table 2). At 0.8 m/s, AX6 and Fitbit had the smallest RE (0.07), followed by Vivofit (0.27), Omron (0.5) and Fenix (0.62), with the AX6 achieving a slightly smaller SD than the Fitbit (0.29 vs. 0.32). At 1 m/s, the Fitbit had the smallest RE (0.08), followed by the AX6, Vivofit, Omron and Fenix.

**Table 2:** RE as mean and SD values for each sensor type and gait speed.

	Speed					
	0.6 m/s		0.8 m/s		1.0 m/s	
Sensor	Mean	SD	Mean	SD	Mean	SD
AX6	−0.02	0.35	0.07	0.29	0.13	0.31
Fitbit	0.21	0.47	0.07	0.32	0.08	0.35
Fenix	0.87	0.24	0.62	0.41	0.48	0.43
Omron	0.92	0.19	0.50	0.40	0.19	0.32
Vivofit	0.57	0.35	0.27	0.38	0.17	0.35

Abbreviations: RE, relative error; SD, standard deviation.

When the sensor was placed on the hand, the Fitbit provided the smallest RE (0.22), followed by AX6 (0.24), Vivofit (0.59), Omron (0.64) and Fenix (0.88) (Table 3). At the hip, the AX6 showed the smallest RE (−0.06), followed by Vivofit (0.2), Fitbit (0.24), Omron (0.44) and Fenix (0.66). Finally, at the ankle, the AX6 again delivered the smallest RE (−0.01), followed by Fitbit (‐0.08), Vivofit (0.27), Fenix (0.46) and Omron (0.65).

**Table 3:** RE as mean and SD values for each sensor type and its position.

	Position of sensor					
	Hand		Hip		Ankle	
Sensor	Mean	SD	Mean	SD	Mean	SD
AX6	0.24	0.47	−0.06	0.18	−0.01	0.08
Fitbit	0.22	0.47	0.24	0.37	−0.08	0.19
Fenix	0.88	0.23	0.66	0.39	0.46	0.43
Omron	0.64	0.37	0.44	0.44	0.65	0.43
Vivofit	0.59	0.39	0.20	0.31	0.27	0.37

Abbreviations: RE, relative error; SD, standard deviation.

### Trial failure

In some cases, the automated measurement did not report any value for the number of gait cycles. This occurred in 41.9% of cases for the Fenix, 34.4% for the Omron, 13.3% for the Vivofit, 8.5% for the FitBit and 8.9% for the AX6.

## DISCUSSION

Step count can serve as a suitable parameter for objective monitoring of rehabilitation progress [10, 14, 28, 44, 49]. However, most of the studies include healthy subjects and little research has been conducted into how commercial wearables behave in the early post‐operative period. Furthermore, gait quality parameters recorded with accelerometers can provide additional, objective information regarding the rehabilitation progress of orthopaedic patients [41, 53]. Objectifiable parameters are of great importance, not least because they do not necessarily correlate with corresponding patient‐reported outcome measurements (PROMs) [35, 62]. The aim of the study was to determine the most accurate way to assess the number of steps taken by orthopaedic patients in the early post‐operative period. The method had to be practical and cost‐effective. This was done by simulating the conditions of this state as accurately as possible.

Previous studies have shown that commercial wearables produce inaccurate measurements for slow walking, partial loading, the use of walking aids or a combination of these factors, as well as depending on the position of the wearables [11, 13, 19, 39, 47, 55, 56, 60, 61]. The faster the walking speed, the more accurate the results [27, 57, 60].

Our study also reflects these results: In terms of speed, all wearables used showed increasing inaccuracy with decreasing speed, with a tendency to underestimate the number of steps taken. All wearables performed best at 1 m/s; the fastest speed tested. The accelerometer (AX6) showed a contrary behaviour in terms of accuracy regarding walking speed. Among the wearables in our study, only the Fitbit showed a somewhat acceptable accuracy (RE 0.21) at 0.6 m/s, the slowest speed tested. However, slow walking is to be expected in the early post‐operative period. Many studies have shown that numerous wearables, including Fitbit, Omron and Garmin devices, do not provide accurate data at slow speeds of 0.2–1.5 m/s [5, 42, 45, 51, 59]. In a systematic review of the general population by Germini et al., the Fitbit Charge had a mean absolute percentage error (MAPE) of <25% in 65% of studies [21]. In another systematic review of the general population, Fitbit and Garmin devices were accurate nearly 50% of the time [20]. In a collective of 258 patients, an average MAPE of 40% was found at speeds of 0.22–0.89 m/s [42]. A RE of −0.02 was observed with the AX6 at 0.6 m/s. Clarke et al. used the AX3 accelerometer, which works similarly to the AX6, in an elderly functionally impaired, walker‐dependent group. They concluded that the AX3 is a valid tool for this patient group [8]. Feng et al. compared 3 accelerometers at speeds ranging from 0.9 to 1.3 m/s. They also showed that accelerometers tend to underestimate steps at low speeds. The AX3 provided the most accurate results of all the accelerometers. With the algorithm used, the authors achieved a percentage error of 22.4% [17].

Our study provides new insights in this area by demonstrating that even at the slow speed at which orthopaedic patients initially move post‐operatively, the number of steps can be accurately determined using an accelerometer with an appropriate algorithm.

Sensor positioning also had a significant influence on accuracy. In our study, the best results were obtained when the AX6 and Fitbit were placed on the ankle (RE −0.01 vs. −0.08). The best position for the Finix was also the ankle, but with an RE of 0.46. The best position for the Vivofit and Omron was the hip with RE of 0.2 and 0.44. McCullagh et al., who placed one accelerometer on the ankle, another on the thigh and also used a pedometer in elderly and frail patients, who were partially dependent on walking aids, only obtained acceptable results with the ankle‐worn accelerometer [38]. A study investigating accelerometer accuracy in older adults, who tend to walk slower, also concluded that the ankle is the best position [45]. This was also shown in other studies regarding the positioning of the Fitbit [16, 52]. For slow walking speeds, the ankle and thigh were found to be the best measurement points in a study of 258 patients [42]. However, Martinato et al. support the use of wrist‐worn devices in elderly populations, as they found a good level of accuracy. Yet, the participants used their preferred walking speed without walking aids [37]. Furthermore, in our study, with the exception of Fitbit, where hand vs hip had a very small difference (RE 0.22 vs. 0.24), positioning on the hip delivered better results than positioning on the hand. This was also observed by Kooner et al., who compared two Fitbit devices, one for hip positioning and one for wrist positioning and by Simpson et al. who placed a Fitbit device at the waist and ankle during slow walking speeds [30, 51]. However, both studies used different Fitbit devices than in our study. Chow et al. showed that waist‐worn Fitbit devices performed better than wrist‐worn devices and that increased speed improved accuracy. Wrist‐worn devices like the Vivofit achieved a MAPE of 22.6% at 0.6 m/s in the study of Mora‐Gonzalez et al. [7, 42].

According to our results, the ankle should be used to assess steps in early post‐operative conditions in orthopaedic patients.

Walking aids are frequently used in orthopaedic patients in the early post‐operative period after operations on the lower extremities and can significantly distort step measurements [13, 30]. This complicates the post‐operative monitoring of the rehabilitation progress.

We observed the same in our study: only the AX6 and Fitbit were able to show consistent results with the walking aids. When using crutches, only the AX6 and Fitbit were able to achieve acceptable measurements with the modified three‐point gait (RE −0.03 vs. 0.05) and two‐point gait (RE −0.08 vs. −0.02). This also occurred when using the walking frame, where only the AX6 and Fitbit achieved a RE of <0.1 (RE −0.03 vs. 0.01). The results using the rolling walker were also striking. All wearables and the accelerometer delivered the least accurate results here, with the AX6 and Fitbit outperforming all devices, but only the AX6 staying below an RE of 0.4 (RE 0.33 vs. 0.42). Compared to the Fitbit the AX6 had a lower SD for all walking aids. This is probably due to the very low excursion of the devices, since on the one hand, there is hardly any excursion of the upper extremities when using the rolling walker, and on the other hand, most of the body weight is leaning against the rolling walker. Lipperts et al. also used a three‐dimensional accelerometer to investigate the daily activities of patients who underwent unicompartmental knee arthroplasty. The study concludes that sufficient monitoring is possible. However, the trial did not take place in the early post‐operative period [33]. Laarhoven et al. also investigated the accuracy of an accelerometer for post‐operative monitoring of total knee arthroplasty patients who used crutches. A mean percentage difference of approx. 7% was found regarding step count measurement. However, the gait type while using crutches was not reported in the study [32]. In addition, no walking speed was measured. In the studies mentioned at the beginning of the discussion [43, 56, 60], the walking speed was also chosen by the participants themselves and therefore did not correspond to the performance of orthopaedic patients immediately after surgery. De Ridder et al. evaluated the Vivofit 3 among others for walking with crutches and advising the participants to perform partial weight‐bearing with 50% as in our study, though this was not controlled, three‐point gait was not performed, and participants chose their own speed. He concluded that the Vivofit 3 was insufficient for counting steps with crutches [13]. In an inpatient rehabilitation setting with an average speed of 0.4 m/s, 32 of the 160 patients used a cane and 79 used a walker. The agreement between Vivofit and Fitbit Charge was 29% and 52%, respectively. Surprisingly, for leg‐worn devices, there was similar accuracy with and without walking aids, but the authors do not present these results in detail [60]. In another study with 25 cane users and 24 walker users, Omron and Fitbit devices underestimated steps by 11.5% [18]. Comparing the Vivofit 2 among others to video step count, no ±20% accuracy could be observed with walking aids and there was a tendency to undercount [36]. Rozanski et al. compared wearables such as the Fitbit Charge HR with an accelerometer in stroke patients. When using a rolling walker, there was no correlation between the wearables and the accelerometer and steps were undercounted [48]. In another stud,y 49% of the participants used a cane or rolling walker. The average speed was 0.7 m/s. The Fitbit Charge had an accuracy of 12.93%, the Omron HJ321 of 58.99% and the Vivofit 18.33% compared to 90.04% for the accelerometer used [26]. Our findings regarding walking aids are reflected in the relevant literature. We were able to show that accelerometers are best suited for step counting when using the most used walking aids in the early post‐operative period and that wearables should be used rather cautiously for this purpose.

Nevertheless, our study has some limitations. In general, the heterogeneity of study designs makes comparisons in this area difficult. In addition, there is great heterogeneity in the wearables used, in part because companies are constantly developing successor models. The small number of participants and the fact that the study involved a healthy group should certainly be mentioned here. Although knee arthroplasty patients are usually able to bear full weight after surgery, they often do not do so in the first few days. However, partial weight‐bearing was simulated in our study. Although it was practised before the gait cycles, it could not be controlled during walking. We also did not test for intermittent walking, which is likely to be common after surgery. Our algorithm was not complex and certainly needs to be optimised for sufficient clinical and ambulatory use. This is particularly evident in the slight overestimation of the actual number of steps taken by the AX6. Asymmetric double stance times and step times can occur during two‐point and modified three‐point gait and vertical acceleration can be disproportionately increased when using walking aids [54]. This could potentially contribute to incorrect measurements. There were also technical problems with the AX6 measurements on three occasions, meaning that not all AX6 cycles could be evaluated.

In summary, we were able to confirm our hypothesis: the use of the AX6 with a rather simple algorithm was nevertheless able to achieve a sufficient step count measurement when simulating the early post‐operative conditions of orthopaedic patients and when using all tested walking aids except the rolling walker. There was also a large difference to the second‐best wearable, the Fitbit, which had a RE of 0.21 at 0.6 m/s. The best sensor position turned out to be the ankle. Accurate post‐operative determination of the number of steps offers new possibilities for objectifying the post‐operative progress, as current PROMs and objective joint function do not correlate with each other [35, 41, 62]. In addition, the patient can receive direct feedback regarding their progress, which also boosts their motivation [2, 63]. Nevertheless, standardisation of the examination conditions is necessary to improve the use of these devices in everyday clinical practice and outpatient care [6]. An EFORT review on accelerometer‐based activity monitoring in orthopaedics concludes that monitoring offers many advantages and will become increasingly important in the future [53].

## CONCLUSION

In conclusion, commercial wearables appear to be of limited use or unsuitable for step count measurement for early post‐operative step count monitoring, as specially modified three‐point and two‐point gait on crutches are frequently used in this early post‐operative period or even represent a discharge criterion. Depending on the model, they can also be quite expensive. Accelerometers, on the other hand, are inexpensive and suitable for step count detection under the same conditions with little effort and could therefore be used in the early post‐operative period to monitor the rehabilitation progress. However, further studies using more complex algorithms are needed on orthopaedic patients to optimise accelerometers for clinical and ambulatory use.

## AUTHOR CONTRIBUTIONS

**Spiros Tsamassiotis**: Conceptualisation; methodology; project administration; supervision; writing – original draft; writing—review and editing. **Michael Schwarze**: Conceptualisation; formal analysis; methodology; visualisation; writing—original draft; writing—review and editing. **Philipp Gehring**: Conceptualisation; investigation; visualisation; software; writing—original draft. **Roman F. Karkosch**: Conceptualisation; visualisation; writing—review and editing. **Lars‐René Tücking**: Conceptualisation; visualisation; writing—review and editing. **Ann‐Kathrin Einfeldt**: Conceptualisation; methodology; investigation. **Eike Jakubowitz**: Conceptualisation; methodology; project administration; supervision; writing—original draft; writing—review and editing. All authors read and approved the final manuscript.

## CONFLICT OF INTEREST STATEMENT

The authors declare no conflicts of interest.

## ETHICS STATEMENT

The study was conducted in accordance with the Declaration of Helsinki and approved by the local Ethics Committee of the Hannover Medical School (No. 9516). Informed consent was obtained from all individual participants included in the study.

## Data Availability

The data that support the findings of this study are available from the corresponding author upon reasonable request.

## References

[1] Ahanathapillai, V. , Amor, J.D. & James, C.J. (2015) Assistive technology to monitor activity, health and wellbeing in old age: the wrist wearable unit in the USEFIL project. Technology and Disability, 27, 17–29. Available from: 10.3233/TAD-150425

[2] Amin, T. , Mobbs, R.J. , Mostafa, N. , Sy, L.W. & Choy, W.J. (2021) Wearable devices for patient monitoring in the early postoperative period: a literature review. mHealth, 7, 50. Available from: 10.21037/mhealth-20-131 34345627 PMC8326951

[3] Aprato, A. , Bechis, M. , Buzzone, M. , Bistolfi, A. , Daghino, W. & Massè, A. (2020) No rest for elderly femur fracture patients: early surgery and early ambulation decrease mortality. Journal of Orthopaedics and Traumatology, 21, 12. Available from: 10.1186/s10195-020-00550-y 32862297 PMC7456623

[4] Bahl, J.S. , Nelson, M.J. , Taylor, M. , Solomon, L.B. , Arnold, J.B. & Thewlis, D. (2018) Biomechanical changes and recovery of gait function after total hip arthroplasty for osteoarthritis: a systematic review and meta‐analysis. Osteoarthritis and Cartilage, 26, 847–863. Available from: 10.1016/j.joca.2018.02.897 29474993

[5] Beevi, F.H.A. , Miranda, J. , Pedersen, C.F. & Wagner, S. (2016) An evaluation of commercial pedometers for monitoring slow walking speed populations. Telemedicine and e‐Health, 22, 441–449. Available from: 10.1089/tmj.2015.0120 26451900

[6] Bunn, J. , Navalta, J.W. , Fountaine, C.J. & Reece, J. (2018) Current state of commercial wearable technology in physical activity monitoring 2015‐2017. International Journal of Exercise Science, 11, 503–515. Available from: 10.70252/NJQX2719 29541338 PMC5841672

[7] Chow, J.J. , Thom, J.M. , Wewege, M.A. , Ward, R.E. & Parmenter, B.J. (2017) Accuracy of step count measured by physical activity monitors: The effect of gait speed and anatomical placement site. Gait & Posture, 57, 199–203. Available from: 10.1016/j.gaitpost.2017.06.012 28666177

[8] Clarke, C.L. , Taylor, J. , Crighton, L.J. , Goodbrand, J.A. , McMurdo, M.E.T. & Witham, M.D. (2017) Validation of the AX3 triaxial accelerometer in older functionally impaired people. Aging Clinical and Experimental Research, 29, 451–457. Available from: 10.1007/s40520-016-0604-8 27435918 PMC5445187

[9] Cristescu, I. , Iordache, D. & Tirlea, C. (2022) Behavioral intention to use smartwatches: a case study. In: *2022 14th International Conference on Electronics, Computers and Artificial Intelligence (ECAI)*, Ploiesti, Romania, 2022, pp. 1–4. Available from: 10.1109/ECAI54874.2022.9847469

[10] Crizer, M.P. , Kazarian, G.S. , Fleischman, A.N. , Lonner, J.H. , Maltenfort, M.G. & Chen, A.F. (2017) Stepping toward objective outcomes: a prospective analysis of step count after total joint arthroplasty. The Journal of Arthroplasty, 32, S162–S165. Available from: 10.1016/j.arth.2017.02.058 28343831

[11] Crouter, S.E. , Schneider, P.L. , Karabulut, M. & Bassett Jr., D.R. (2003) Validity of 10 electronic pedometers for measuring steps, distance, and energy cost. Medicine and Science in Sports and Exercise, 35, 1455–1460. Available from: 10.1249/01.Mss.0000078932.61440.A2 12900704

[12] Cyarto, E.V. , Myers, A. & Tudor‐Locke, C. (2004) Pedometer accuracy in nursing home and community‐dwelling older adults. Medicine and Science in Sports and Exercise, 36, 205–209. Available from: 10.1249/01.Mss.0000113476.62469.98 14767241

[13] De Ridder, R. & De Blaiser, C. (2019) Activity trackers are not valid for step count registration when walking with crutches. Gait & Posture, 70, 30–32. Available from: 10.1016/j.gaitpost.2019.02.009 30798092

[14] Del Din, S. , Hickey, A. , Ladha, C. , Stuart, S. , Bourke, A.K. , Esser, P. et al. (2016) Instrumented gait assessment with a single wearable: an introductory tutorial. F1000Research, 5, 2323. Available from: 10.12688/f1000research.9591.1

[15] Demir, Y.P. & Yildirim, S.A. (2019) Different walk aids on gait parameters and kinematic analysis of the pelvis in patients with Adult Neuromuscular Disease. Neurosciences, 24, 36–44. Available from: 10.17712/nsj.2019.1.20180316 30842398 PMC8015543

[16] Feehan, L.M. , Geldman, J. , Sayre, E.C. , Park, C. , Ezzat, A.M. , Yoo, J.Y. et al. (2018) Accuracy of Fitbit devices: systematic review and narrative syntheses of quantitative data. JMIR mHealth and uHealth, 6, e10527. Available from: 10.2196/10527 30093371 PMC6107736

[17] Feng, Y. , Wong, C.K. , Janeja, V. , Kuber, R. & Mentis, H.M. (2017) Comparison of tri‐axial accelerometers step‐count accuracy in slow walking conditions. Gait & Posture, 53, 11–16. Available from: 10.1016/j.gaitpost.2016.12.014 28064084

[18] Floegel, T.A. , Florez‐Pregonero, A. , Hekler, E.B. & Buman, M.P. (2017) Validation of consumer‐based hip and wrist activity monitors in older adults with varied ambulatory abilities. The Journals of Gerontology Series A: Biological Sciences and Medical Sciences, 72, 229–236. Available from: 10.1093/gerona/glw098 27257217 PMC6082588

[19] Fokkema, T. , Kooiman, T.J.M. , Krijnen, W.P. , Van Der Schans, C.P. & De Groot, M. (2017) Reliability and validity of ten consumer activity trackers depend on walking speed. Medicine & Science in Sports & Exercise, 49, 793–800. Available from: 10.1249/mss.0000000000001146 28319983

[20] Fuller, D. , Colwell, E. , Low, J. , Orychock, K. , Tobin, M.A. , Simango, B. et al. (2020) Reliability and validity of commercially available wearable devices for measuring steps, energy expenditure, and heart rate: systematic review. JMIR mHealth and uHealth, 8, e18694. Available from: 10.2196/18694 32897239 PMC7509623

[21] Germini, F. , Noronha, N. , Borg Debono, V. , Abraham Philip, B. , Pete, D. , Navarro, T. et al. (2022) Accuracy and acceptability of wrist‐wearable activity‐tracking devices: systematic review of the literature. Journal of Medical Internet Research, 24, e30791. Available from: 10.2196/30791 35060915 PMC8817215

[22] Glattke, K.E. , Tummala, S.V. & Chhabra, A. (2022) Anterior cruciate ligament reconstruction recovery and rehabilitation: a systematic review. Journal of Bone and Joint Surgery, 104, 739–754. Available from: 10.2106/jbjs.21.00688 34932514

[23] Halfwerk, F.R. , van Haaren, J.H.L. , Klaassen, R. , van Delden, R.W. , Veltink, P.H. & Grandjean, J.G. (2021) Objective quantification of in‐hospital patient mobilization after cardiac surgery using accelerometers: selection, use, and analysis. Sensors, 21, 1979. Available from: 10.3390/s21061979 33799717 PMC7999757

[24] Hansen, T.B. , Gromov, K. , Kristensen, B.B. , Husted, H. & Kehlet, H. (2017) [Fast‐track hip arthroplasty]. Ugeskrift For Laeger, 179, 29260691. 29260691

[25] Henriksen, A. , Haugen Mikalsen, M. , Woldaregay, A.Z. , Muzny, M. , Hartvigsen, G. , Hopstock, L.A. et al. (2018) Using fitness trackers and smartwatches to measure physical activity in research: analysis of consumer wrist‐worn wearables. Journal of Medical Internet Research, 20, e110. Available from: 10.2196/jmir.9157 29567635 PMC5887043

[26] Hergenroeder, A.L. , Barone Gibbs, B. , Kotlarczyk, M.P. , Perera, S. , Kowalsky, R.J. & Brach, J.S. (2019) Accuracy and acceptability of commercial‐grade physical activity monitors in older adults. Journal of Aging and Physical Activity, 27, 222–229. Available from: 10.1123/japa.2018-0036 30117355 PMC7023914

[27] Holbrook, E.A. , Barreira, T.V. & Kang, M. (2009) Validity and reliability of Omron pedometers for prescribed and self‐paced walking. Medicine & Science in Sports & Exercise, 41, 670–674. Available from: 10.1249/MSS.0b013e3181886095 19204582

[28] Höll, S. , Blum, A. , Gosheger, G. , Dieckmann, R. , Winter, C. & Rosenbaum, D. (2018) Clinical outcome and physical activity measured with StepWatch 3™ Activity Monitor after minimally invasive total hip arthroplasty. Journal of Orthopaedic Surgery and Research, 13, 148. Available from: 10.1186/s13018-018-0775-4 29907134 PMC6003151

[29] Jung, K.D. , Husted, H. & Kristensen, B.B. (2020) Knie‐ und Hüfttotalendoprothese in 2 Tagen: Das dänische Fast‐Track‐Modell. Der Orthopäde, 49, 218–225. Available from: 10.1007/s00132-019-03796-5 31451893

[30] Kooner, P. , Schubert, T. , Howard, J.L. , Lanting, B.A. , Teeter, M.G. & Vasarhelyi, E.M. (2021) Evaluation of the effect of gait aids, such as canes, crutches, and walkers, on the accuracy of step counters in healthy individuals. Orthopedic Research and Reviews, 13, 1–8. Available from: 10.2147/orr.S292255 33447097 PMC7802358

[31] Kort, N.P. & Clarius, M. (2018) Fast track in TKA surgery: where are we now? In: Kerkhoffs, G.M.M.J. , Haddad, F. , Hirschmann, M.T. , Karlsson, J. & Seil, R. (Eds.) ESSKA Instructional Course Lecture Book: Glasgow 2018. Berlin, Heidelberg: Springer Berlin Heidelberg, pp. 81–84. Available from 10.1007/978-3-662-56127-0_6

[32] van Laarhoven, S.N. , Lipperts, M. , Bolink, S.A.A.N. , Senden, R. , Heyligers, I.C. & Grimm, B. (2016) Validation of a novel activity monitor in impaired, slow‐walking, crutch‐supported patients. Annals of Physical and Rehabilitation Medicine, 59, 308–313. Available from: 10.1016/j.rehab.2016.05.006 27659237

[33] Lipperts, M. , van Laarhoven, S. , Senden, R. , Heyligers, I. & Grimm, B. (2017) Clinical validation of a body‐fixed 3D accelerometer and algorithm for activity monitoring in orthopaedic patients. Journal of Orthopaedic Translation, 11, 19–29. Available from: 10.1016/j.jot.2017.02.003 29662766 PMC5866408

[34] Lu, T.C. , Fu, C.M. , Ma, M. , Fang, C.C. & Turner, A. (2016) Healthcare applications of smart watches. a systematic review. Applied Clinical Informatics, 7, 850–869. Available from: 10.4338/aci-2016-03-r-0042 27623763 PMC5052554

[35] Luna, I.E. , Kehlet, H. , Peterson, B. , Wede, H.R. , Hoevsgaard, S.J. & Aasvang, E.K. (2017) Early patient‐reported outcomes versus objective function after total hip and knee arthroplasty: a prospective cohort study. The Bone & Joint Journal, 99–b, 1167–1175. Available from: 10.1302/0301-620x.99b9.Bjj-2016-1343.R1 28860396

[36] Madigan, E.A. (2019) Fitness band accuracy in older community dwelling adults. Health Informatics Journal, 25, 676–682. Available from: 10.1177/1460458217720399 28743215

[37] Martinato, M. , Lorenzoni, G. , Zanchi, T. , Bergamin, A. , Buratin, A. , Azzolina, D. et al. (2021) Usability and accuracy of a smartwatch for the assessment of physical activity in the elderly population: observational study. JMIR mHealth and uHealth, 9, e20966. Available from: 10.2196/20966 33949953 PMC8135023

[38] McCullagh, R. , Dillon, C. , O'Connell, A.M. , Horgan, N.F. & Timmons, S. (2017) Step‐count accuracy of 3 motion sensors for older and frail medical inpatients. Archives of Physical Medicine and Rehabilitation, 98, 295–302. Available from: 10.1016/j.apmr.2016.08.476 27666157

[39] Melanson, E.L. , Knoll, J.R. , Bell, M.L. , Donahoo, W.T. , Hill, J.O. , Nysse, L.J. et al. (2004) Commercially available pedometers: considerations for accurate step counting. Preventive Medicine, 39, 361–368. Available from: 10.1016/j.ypmed.2004.01.032 15226047

[40] Mendel, T. , Schenk, P. , Ullrich, B.W. , Hofmann, G.O. , Goehre, F. , Schwan, S. et al. (2021) Mid‐term outcome of bilateral fragility fractures of the sacrum after bisegmental transsacral stabilization versus spinopelvic fixation. The Bone & Joint Journal, 103–b, 462–468. Available from: 10.1302/0301-620x.103b3.Bjj-2020-1454.R1 33641427

[41] Mills, K. , Falchi, B. , Duckett, C. & Naylor, J. (2019) Minimal change in physical activity after lower limb joint arthroplasty, but the outcome measure may be contributing to the problem: a systematic review and meta‐analysis. Physiotherapy, 105, 35–45. Available from: 10.1016/j.physio.2018.04.003 30025714

[42] Mora‐Gonzalez, J. , Gould, Z.R. , Moore, C.C. , Aguiar, E.J. , Ducharme, S.W. , Schuna, J.M. et al. (2022) A catalog of validity indices for step counting wearable technologies during treadmill walking: the CADENCE‐adults study. International Journal of Behavioral Nutrition and Physical Activity, 19, 117. Available from: 10.1186/s12966-022-01350-9 36076265 PMC9461139

[43] Nakagata, T. , Murakami, H. , Kawakami, R. , Tripette, J. , Nakae, S. , Yamada, Y. et al. (2022) Step‐count outcomes of 13 different activity trackers: results from laboratory and free‐living experiments. Gait & Posture, 98, 24–33. Available from: 10.1016/j.gaitpost.2022.08.004 36030707

[44] North, K. , Simpson, G.M. , Stuart, A.R. , Kubiak, E.N. , Petelenz, T.J. , Hitchcock, R.W. et al. (2023) Early postoperative step count and walking time have greater impact on lower limb fracture outcomes than load‐bearing metrics. Injury, 54, 110756. Available from: 10.1016/j.injury.2023.04.043 37202224

[45] Phillips, L.J. , Petroski, G.F. & Markis, N.E. (2015) A comparison of accelerometer accuracy in older adults. Research in Gerontological Nursing, 8, 213–219. Available from: 10.3928/19404921-20150429-03 25942386

[46] R Core Team (2021) *R: a language and environment for statistical computing*. Vienna, Austria: R Foundation for Statistical Computing. Available from: https://www.R-project.org/

[47] Rosenberger, M.E. , Buman, M.P. , Haskell, W.L. , McConnell, M.V. & Carstensen, L.L. (2016) Twenty‐four hours of sleep, sedentary behavior, and physical activity with nine wearable devices. Medicine & Science in Sports & Exercise, 48, 457–465. Available from: 10.1249/mss.0000000000000778 26484953 PMC4760880

[48] Rozanski, G.M. , Aqui, A. , Sivakumaran, S. & Mansfield, A. (2018) Consumer wearable devices for activity monitoring among individuals after a stroke: a prospective comparison. JMIR Cardio, 2, e1. Available from: 10.2196/cardio.8199 31758760 PMC6834221

[49] Schotanus, M.G.M. , Bemelmans, Y.F.L. , Grimm, B. , Heyligers, I.C. & Kort, N.P. (2017) Physical activity after outpatient surgery and enhanced recovery for total knee arthroplasty. Knee Surgery, Sports Traumatology, Arthroscopy, 25, 3366–3371. Available from: 10.1007/s00167-016-4256-1 27492381

[50] Seifert, A. (2020) Smartwatch use among older adults: findings from two large surveys. In: *Human Aspects of IT for the Aged Population. Technologies, Design and User Experience: 6th International Conference, ITAP 2020, Held as Part of the 22nd HCI International Conference, HCII 2020, Proceedings, Part I*, Copenhagen, Denmark, 19–24 July 2020.

[51] Simpson, L. , Eng, J. , Klassen, T. , Lim, S. , Louie, D. , Parappilly, B. et al. (2015) Capturing step counts at slow walking speeds in older adults: comparison of ankle and waist placement of measuring device. Journal of Rehabilitation Medicine, 47, 830–835. Available from: 10.2340/16501977-1993 26181670

[52] Singh, A.K. , Farmer, C. , Van Den Berg, M.L.E. , Killington, M. & Barr, C.J. (2016) Accuracy of the FitBit at walking speeds and cadences relevant to clinical rehabilitation populations. Disability and Health Journal, 9, 320–323. Available from: 10.1016/j.dhjo.2015.10.011 26905972

[53] Sliepen, M. , Lipperts, M. , Tjur, M. & Mechlenburg, I. (2019) Use of accelerometer‐based activity monitoring in orthopaedics: benefits, impact and practical considerations. EFORT Open Reviews, 4, 678–685. Available from: 10.1302/2058-5241.4.180041 32010456 PMC6986392

[54] Smidt, G.L. & Mommens, M.A. (1980) System of reporting and comparing influence of ambulatory aids on gait. Physical Therapy, 60, 551–558. Available from: 10.1093/ptj/60.5.551 7384225

[55] Storti, K.L. , Pettee, K.K. , Brach, J.S. , Talkowski, J.B. , Richardson, C.R. & Kriska, A.M. (2008) Gait speed and step‐count monitor accuracy in community‐dwelling older adults. Medicine and Science in Sports and Exercise, 40, 59–64. Available from: 10.1249/mss.0b013e318158b504 18091020

[56] Tedesco, S. , Sica, M. , Ancillao, A. , Timmons, S. , Barton, J. & O'Flynn, B. (2019) Validity evaluation of the Fitbit Charge2 and the Garmin vivosmart HR+ in free‐living environments in an older adult cohort. JMIR mHealth and uHealth, 7, e13084. Available from: 10.2196/13084 31219048 PMC6607774

[57] Tedesco, S. , Sica, M. , Ancillao, A. , Timmons, S. , Barton, J. & O'Flynn, B. (2019) Accuracy of consumer‐level and research‐grade activity trackers in ambulatory settings in older adults. PLoS One, 14, e0216891. Available from: 10.1371/journal.pone.0216891 31112585 PMC6529154

[58] Thompson, W. (2016) Worldwide survey of fitness trends for 2017. ACSM's Health & Fitness Journal, 20, 8–17. Available from: 10.1249/FIT.0000000000000252

[59] Tophøj, K.H. , Petersen, M.G. , Sæbye, C. , Baad‐Hansen, T. & Wagner, S. (2018) Validity and reliability evaluation of four commercial activity trackers' step counting performance. Telemedicine and e‐Health, 24, 669–677. Available from: 10.1089/tmj.2017.0264 29303680

[60] Treacy, D. , Hassett, L. , Schurr, K. , Chagpar, S. , Paul, S.S. & Sherrington, C. (2017) Validity of different activity monitors to count steps in an inpatient rehabilitation setting. Physical Therapy, 97, 581–588. Available from: 10.1093/ptj/pzx010 28339904

[61] Tudor‐Locke, C. , Ainsworth, B.E. , Thompson, R.W. & Matthews, C.E. (2002) Comparison of pedometer and accelerometer measures of free‐living physical activity. Medicine and Science in Sports and Exercise, 34, 2045–2051. Available from: 10.1097/00005768-200212000-00027 12471314

[62] Vaughn, N.H. , Dunklebarger, M.F. & Mason, M.W. (2019) Individual patient‐reported activity levels before and after joint arthroplasty are neither accurate nor reproducible. Clinical Orthopaedics & Related Research, 477, 536–544. Available from: 10.1097/corr.0000000000000591 30543533 PMC6382186

[63] Weakley, J. , Cowley, N. , Schoenfeld, B.J. , Read, D.B. , Timmins, R.G. , García‐Ramos, A. et al. (2023) The effect of feedback on resistance training performance and adaptations: a systematic review and meta‐analysis. Sports Medicine, 53, 1789–1803. Available from: 10.1007/s40279-023-01877-2 37410360 PMC10432365

[64] Yüksel, İ. & Kinikli, G.İ. (2012) Early rehabilitation after surgery. In: Doral, M.N. (Ed.) Sports injuries: Prevention, diagnosis, treatment and rehabilitation. Berlin, Heidelberg: Springer Berlin Heidelberg, pp. 1127–1130. Available from 10.1007/978-3-642-15630-4_150

